# VALIDATION OF THE PITTSBURGH PERFORMANCE FATIGABILITY INDEX FROM USUAL-PACED 400 M WALK

**DOI:** 10.1093/geroni/igac059.1455

**Published:** 2022-12-20

**Authors:** Yujia (Susanna) Qiao, Jaroslaw Harzelak, Pamela Toto, Kyle Moored, Bret Goodpaster, Adam Santanasto, Barbara Nicklas, Nancy W Glynn

**Affiliations:** University of Pittsburgh, Schoold of Public Health, Pittsburgh, Pennsylvania, United States; Indiana University, School of Public Health in Bloomington, Bloomington, Indiana, United States; University of Pittsburgh, Pittsburgh, Pennsylvania, United States; Johns Hopkins Bloomberg School of Public Health, Baltimore, Maryland, United States; AdventHealth, Orlando, Florida, United States; School of Public Health, University of Pittsburgh, Oakmont, Pennsylvania, United States; Wake Forest School of Medicine, Winston-Salem, North Carolina, United States; School of Public Health, University of Pittsburgh, Pittsburgh, Pennsylvania, United States

## Abstract

The Pittsburgh Performance Fatigability Index (PPFI, higher=greater fatigability), a novel accelerometer-based performance fatigability measure, was recently developed for adults aged ≥60 years. We validated PPFI during a usual-paced 400m walk in 429 individuals enrolled in the Study of Muscle, Mobility and Aging (age=76.9±5.3years, 57.6% women, gait speed=1.0±0.2 m/s from 4-meter walk). PPFI quantifies percent of performance decrement (i.e., slowing down) during 400m walk by comparing area under the observed cadence trajectories to the hypothetical area that reflects sustaining maximum cadence for the entire walk. PPFI scores (mean=2.1%±2.5%, range: 0-21.7%) demonstrated convergent validity with Short Physical Performance Battery (SPPB, Pearson correlation (r)=-0.32) and mobility (time to walk 400m, r=0.47). PPFI scores discriminated higher versus lower physical function (SPPB: 1.6% (≥10) vs. 2.9% (<10), p<.001), adjusted for age, sex, height, and weight. PPFI is the first validated accelerometer-based objective risk assessment tool for measuring performance fatigability, an established marker of functional decline.

